# How Does Increased Human Astrocyte‐Derived α‐Synuclein Affect Trophic Factor Release and Neurodegeneration in Neuron‐Astrocyte Synaptic Components?

**DOI:** 10.1002/alz.090249

**Published:** 2025-01-03

**Authors:** Büşra Şengül, Erdinc Dursun, Duygu Gezen‐Ak

**Affiliations:** ^1^ Institute of Neurological Sciences, Istanbul University‐Cerrahpasa, Istanbul Turkey

## Abstract

**Background:**

Astrocytes secrete neuromodulators, neurohormones, trophic factors, and synaptogenesis modulators. Trophic factors regulate various cellular processes including synaptic transmission. Astrocytes have critical roles in synaptic development and plasticity. They are involved in synapse formation and their absence leads to loss of synaptic connections and, neurodegeneration. Our previous study reported that α‐synuclein overexpression in human astrocytes alters the production and release of trophic factors. In the present study, we investigated the potential of astrocyte‐derived α‐synuclein increment to trigger the degeneration of synaptic connections and promote neurodegeneration by affecting the release of growth factors required for synapse formation.

**Method:**

A co‐culture system containing LUHMES cells which are human precursor neural cells, and human primary astrocyte cells was established. The plasmid carrying the human SNCA gene was transferred only to astrocytes using a lipid‐based transfection agent and then, SNCA overexpressing astrocytes were co‐cultured with neurons. We investigated whether the transfected α‐synuclein in astrocytes transffered to neurons or not by live cell imaging. The levels of synaptic proteins such as thrombospondin, synaptophysin, PSD‐95, and NGF were determined by western blot.

**Result:**

Our data showed that astrocyte‐derived α‐synuclein migrated to LUHMES cells, α‐synuclein pathology increased neuronal cytotoxicity and decreased NGF expression. We found that astrocyte‐derived α‐synuclein increased the production and release of astrocyte‐derived thrombospondin, the production of synaptophysin and PSD‐95 proteins that are all involved in synapse formation in neurons.

**Conclusion:**

Our results indicate that α‐synuclein may affect synapse formation by increasing the thrombospondin levels. Evaluating this result with the increased membrane damage, we may speculate that these increments in synaptic proteins may be considered as a cellular response to reverse the damaged cell structures.

